# Deep intronic founder mutations identified in the *ERCC4*/*XPF* gene are potential therapeutic targets for a high-frequency form of xeroderma pigmentosum

**DOI:** 10.1073/pnas.2217423120

**Published:** 2023-06-26

**Authors:** Chikako Senju, Yuka Nakazawa, Taichi Oso, Mayuko Shimada, Kana Kato, Michiko Matsuse, Mariko Tsujimoto, Taro Masaki, Yasushi Miyazaki, Satoshi Fukushima, Satoshi Tateishi, Atsushi Utani, Hiroyuki Murota, Katsumi Tanaka, Norisato Mitsutake, Shinichi Moriwaki, Chikako Nishigori, Tomoo Ogi

**Affiliations:** ^a^Department of Genetics, Research Institute of Environmental Medicine, Nagoya University, Nagoya 464-8601, Japan; ^b^Department of Human Genetics and Molecular Biology, Graduate School of Medicine, Nagoya University, Nagoya 464-8601, Japan; ^c^Department of Genome Repair, Atomic Bomb Disease Institute, Nagasaki University, Nagasaki 852-8523, Japan; ^d^Department of Radiation Medical Sciences, Atomic Bomb Disease Institute, Nagasaki University, Nagasaki 852-8523, Japan; ^e^Department of Hematology, Atomic Bomb Disease Institute, Nagasaki University, Nagasaki 852-8523, Japan; ^f^Department of Plastic and Reconstructive Surgery, Graduate School of Biomedical Sciences, Nagasaki University, Nagasaki 852-8501, Japan; ^g^Division of Dermatology, Department of Internal Related, Kobe University Graduate School of Medicine, Kobe 650-0017, Japan; ^h^Department of Dermatology and Plastic Surgery, Faculty of Life Sciences, Kumamoto University, Kumamoto 860-8556, Japan; ^i^Department of Cell Maintenance, Institute of Molecular Embryology and Genetics, Kumamoto University, Kumamoto 860-0811, Japan; ^j^Department of Dermatology, Graduate School of Biomedical Sciences, Nagasaki University, Nagasaki 852-8501, Japan; ^k^Leading Medical Research Core Unit, Life-Science Innovation, Graduate School of Biomedical Sciences, Nagasaki University, Nagasaki 852-8501, Japan; ^l^Department of Dermatology, Osaka Medical and Pharmaceutical University, Takatsuki 569-8686, Japan; ^m^Department of iPS cell applications, Graduate School of Medicine, Kobe University, Kobe 650-0017, Japan; ^n^Division of Animal Medical Science, Center for One Medicine Innovative Translational Research, Nagoya University, Nagoya 464-8601, Japan; ^o^Division of Molecular Physiology and Dynamics, Institute for Glyco-core Research, Tokai National Higher Education and Research System, Nagoya 464-8601, Japan

**Keywords:** xeroderma pigmentosum, nucleotide excision repair (NER), artificial antisense oligonucleotides (ASOs), oligonucleotide therapeutics, DNA repair

## Abstract

Xeroderma pigmentosum (XP) is a genodermatosis defined by cutaneous photosensitivity with an increased risk of skin tumors because of DNA repair deficiency. The worldwide prevalence of XP is ~1 to 4 in million, with higher incidence in some countries and regions including Japan (1 in 22,000) and North Africa due to founder mutations and a high degree of consanguinity. Among XP, the complementation group F (XP-F), is a rare form (1% of worldwide XP); however, this is underdiagnosed, because the *ERCC4*/*XPF* gene is essential for fetal development and most of previously reported *ERCC4*/*XPF* pathogenic variants are hypomorphs causing relatively mild phenotypes. From the largest Japanese XP cohort study, we report 17 XP-F cases bearing two pathogenic variants, both identified in deep intronic regions of the *ERCC4*/*XPF* gene. The first variant, located in intron 1, is a Japanese founder mutation, which additionally accounts for ~10% of the entire Japanese XP cases (MAF = 0.00196), causing an aberrant pre-mRNA splicing due to a miss-binding of U1snRNA. The second mutation located in intron eight induces an alternative polyadenylation. Both mutations cause a reduction of the *ERCC4*/*XPF* gene expression, resulting in XP clinical manifestations. Most cases developed early-onset skin cancers, indicating that these variants need critical attention. We further demonstrate that antisense oligonucleotides designed for the mutations can restore the XPF protein expression and DNA repair capacity in the patients’ cells. Collectively, these pathogenic variants can be potential therapeutic targets for XP.

## Significance

Xeroderma pigmentosum (XP) is a genodermatosis that occasionally causes neurological abnormalities because of impaired DNA repair. The XPF endonuclease, compromised in the XP-F complementation group patients, is vital for genome maintenance. Patients completely lacking the XPF functionality are extremely rare and die in early infancy from fatal malformations. Other XP-F cases are still rare (<1% worldwide-XP), but they exhibit milder manifestations with residual XPF actibities. From the largest Japanese XP-cohort study, we identified 17 cases with previously uncharacterized *ERCC4*/*XPF* deep intronic founder mutations. One mutation accounts for approximately 10% of all Japanese XP patients (MAF = 0.002). Most cases developed early-onset skin cancers, necessitating critical attention to these mutations. Artificial antisense-oligonucleotide–based therapeutics hold promise for high-frequency XP cases with intron mutations.

Xeroderma pigmentosum (XP) is a rare genodermatosis associated with a dysfunction of DNA repair systems responsible for the processing of sunlight-induced photolesions (1–5). Typical XP patients display severe skin manifestations, including profound photosensitivity, dry skin, unusual pigmentation, and cancer predisposition of sun-exposed areas, many of which are common to all XP classes; in a minority of cases, they also develop neurological symptoms (6, 7). These XP clinical manifestations are heterogenous and highly dependent on the affected genes and type of mutations, living environment, and the thoroughness of medical interventions (7).

Typical XP cases are classified into seven genetic complementation groups, XP-A to XP-G (classical XP), as well as XP-variant (XP-V), based on the affected genes (*XPA* to *ERCC5*/*XPG*, and *POLH*/*XPV*). Classical XP patients are associated with defective nucleotide excision repair (NER) of photolesions (8–10), while XP variants are NER-proficient but are impaired in bypass synthesis (translesion synthesis: TLS) of damaged DNA (11, 12). Besides these major forms, several cases with specific minor mutations in *ERCC3*/*XPB*, *ERCC2*/*XPD*, and *ERCC5*/*XPG* display the combined features of XP and an orphan progeroid disorder, Cockayne syndrome (CS), termed XPCS (13, 14). In exceptional cases, patients with rare variants in *ERCC4*/*XPF*, encoding a subunit of the ERCC1-XPF NER endonuclease, exhibit the combined phenotypes of XP, CS, and Fanconi anemia (FA), a rare inherited bone marrow failure syndrome (IBMFS), designated XPCSFA (15).

NER removes bulky UV photoproducts and base adducts (16). Global genome NER (GG-NER) handles DNA damage anywhere in the genome (16), while transcription-coupled NER (TC-NER) rapidly removes transcription-blocking lesions from actively transcribed genes (17–20). The damage recognition process of GG-NER is mediated by the DDB2/XPE and XPC proteins (21), whereas TC-NER is initiated by the processing of stalled RNA polymerase II (22–24). After the damage recognition, all other XP proteins are involved in the damage incision process (25, 26). Because of the residual TC-NER activity, XP-C and XP-E patients do not display severe sunburn or neurological symptoms (5).

The incidence of XP is 2 to 4 in million in Western Europe and North America countries, wherein the most prevalent complementation groups are XP-C and XP-A (7). By contrast, in Japan, the incidence is 1 in 22,000 (2, 3); XP-A (53%) and XP-V (31%) account for the majority of the cases because of highly prevalent founder mutations (e.g., *XPA* c.390-1G>C; *POLH* c.490G>T) (27, 28). In contrast, XP-F is a rare form (1% in worldwide; 4% in Japan); however, XP-F cases are underdiagnosed, as typical early-onset patients only display relatively mild skin phenotypes. A limited number of patients with mutations in *ERCC4/XPF* display late-onset neurological symptoms with or without skin cancers (29, 30), although only exceptional cases with rare variants show early onset devastating symptoms observed in CS, FA, XPCSFA, cerebro-oculo-facio-skeletal syndrome (COFS), and XFE progeroid syndrome, because the ERCC1-XPF endonuclease is essential for development (15, 31–35).

In this report, from the largest Japanese XP cohort study, we describe 17 XP-F cases, all having one of two deep-intronic variants of *ERCC4*/*XPF*. Both mutations caused a significant reduction of the XPF protein expression and patients’ cells exhibited DNA repair deficiency. The variants are located in a linkage disequilibrium (LD) block that spans ~55 kbp including the entire *ERCC4*/*XPF* gene. SNP analysis reveals that two unique haplotypes are shared among the patients, suggesting that common ancestral origins for the identified intronic variants underlie the disease development. Note that, the variant in intron 1 is a high-frequency founder mutation, being responsible for ~10% of the entire Japanese XP cases. Remarkably, a treatment of patient cells by antisense oligonucleotides designed for the mutations successfully restored the cellular XPF expression and DNA repair activity. Collectively, our results demonstrate that these variants can be potential therapeutic targets for XP.

## Results

### Case Reports.

**Fig. 1.**
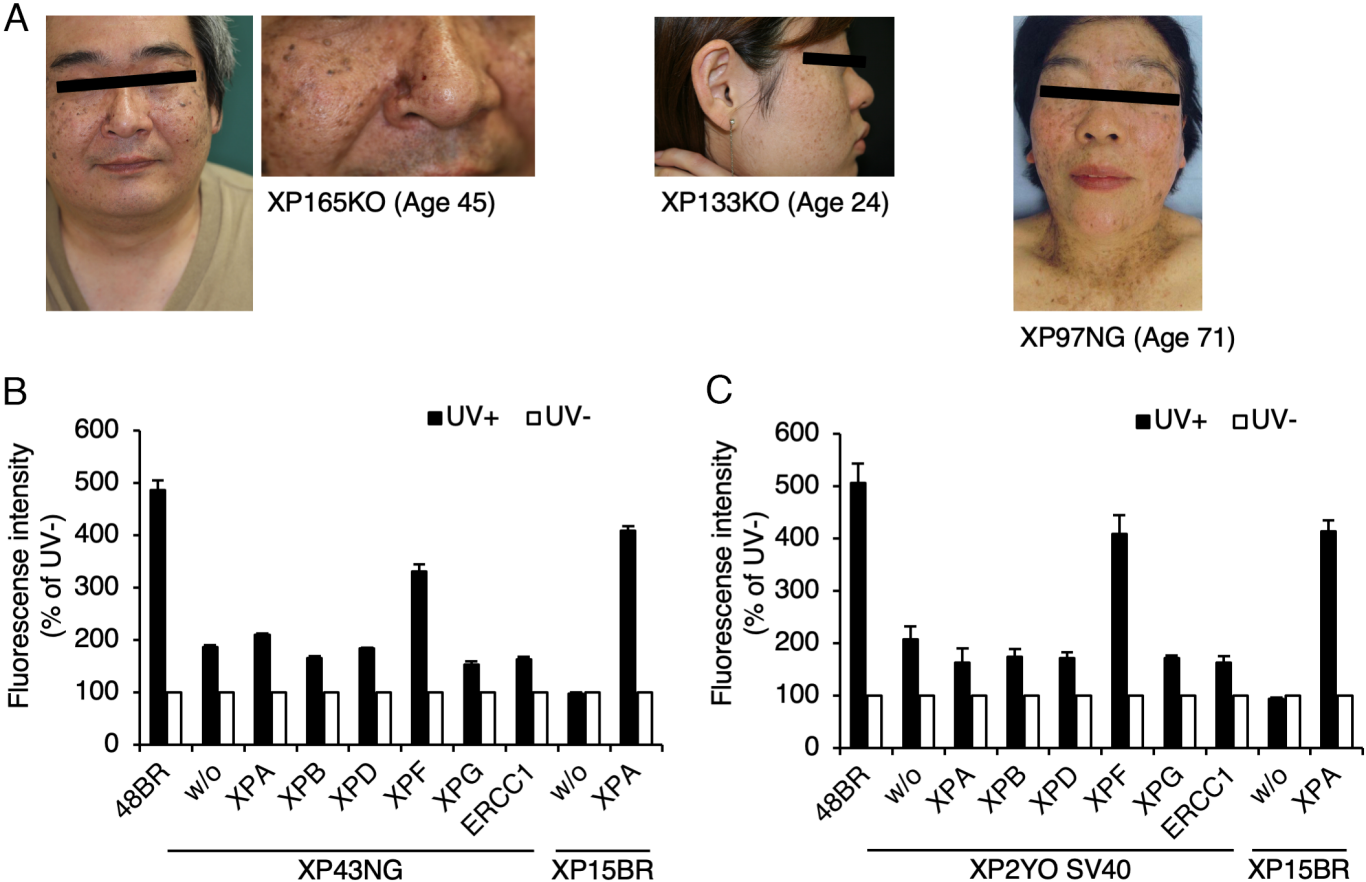
Japanese XP-F cases. (*A*) Photos of representative cases. XP165KO, age 45 (first and second panels); XP133KO, age 24 (third panel); XP97NG, age 71. (*B*) Lentivirus-based complementation assay. Exclusive rescue of the UDS deficiency by the infection of recombinant lentivirus expressing the wild-type *ERCC4*/*XPF* cDNA in XP43NG cells (filled bars, 20 J/m^2^ UVC; open bars, without UV). (*C*) Lentivirus-based complementation assay. XP2YO was also assigned to the XP-F complementation group. UDS was normalized to activity in nonirradiated cells. Error bars: SD of means of at least quintuplicate wells (*B* and *C*, representative data). See also *SI Appendix*, Fig. S1.

Detailed clinical reports of representative cases are described below. As summarized in Table 1, all cases display typical XP-F clinical manifestations; photos of the representative cases are shown in Fig. 1*A*. XP101OS (36), XP4NG (37), XP23OS (38), and XP2YO/XP3YO (39) are published previously as clinical reports. Most of the cases developed early-onset sun sensitivity during their childhood; devastating symptoms such as skin carcinomas were present in their middle age. Varying degrees of photosensitivity in childhood may depend on the daily practice of sunlight protection and geographic conditions. These cases were extracted from the largest Japanese XP cohort study: patients enrolled in the Genome Instability Syndrome Diagnosis Project, a part of the Rare/Intractable Disease (nanbyo) Project of Japan at the Research Institute of Environmental Medicine, Nagoya University; the collections of registered XP-patients in the Departments of Dermatology, Kobe University and the Osaka Medical and Pharmaceutical University. Unscheduled DNA synthesis (UDS) data in Table 1 were extracted from our archives.

**Table 1.** *ERCC4*/*XPF* intron mutations identified in Japanese XP-F patients

Subject ID	Sex	First visit	Onset	*ERCC4*/*XPF* mutations		Clinical findings	UDS %	Reference
				**Allele1**	**Allele2**			
XP136KO	F	44 y	3 y	**intron 1 c.207+196T>A**	**intron 1 c.207+196T>A**	decreased in MED & delayed erythema peak, BCC+, photophobia	13	this study
XP37NG	M	35 y	3 y	**intron 1 c.207+196T>A**	**intron 1 c.207+196T>A**	exposed pigmentation and dry skin, skin cancer	21	this study
XP43NG	F	50 y	severe sunburn from 5 y abnormal pigmented spots from 16 y	**intron 1 c.207+196T>A**	**intron 1 c.207+196T>A**	no skin cancer	21	this study
XP101OS	F	49 y	extensive sunburn from childhood	**intron 1 c.207+196T>A**	**intron 1 c.207+196T>A**	photophobia from childhood, marked photoaged skin on sun-exposed area, MED 30 mJ/cm^2^, BCC (47 y, 49 y), KA (49 y)	31	Ref. 36
XP97NG	F	71 y	no history of severe sunburn abnormal pigmented spots from 16 y	**intron 1 c.207+196T>A**	**intron 1 c.207+196T>A**	no skin cancer	18	this study
XP165KO	M	44 y	severe sunburn from childhood	**intron 1 c.207+196T>A**	**intron 1 c.207+196T>A**	decrease in MED (30 mJ/cm^2^) and delayed erythema peak, multiple BCC (44 y)	19	this study
XP103NG	F	48 y	severe sunburn from 5 y abnormal pigmented spots from 30 y	**intron 1 c.207+196T>A**	**intron 1 c.207+196T>A**	no skin cancer	15	this study
XP4NG	M	64 y	5 y	**intron 1 c.207+196T>A**	exon 11 c.2749T>C p.*917Rext*83	exposed pigmentation and dry skin, decrease in MED (28.0 mJ/cm^2^) and delayed erythema peak, multiple AK	7	Ref. 37
XP48NG	M	67 y	extensive sunburn and pigmented plaque on the sun-exposed area from early childhood	**intron 1 c.207+196T>A**	exon 11 c.2749T>C p.*917Rext*83	multiple BCC and AK (at the first visit)	3	this study
XP90NG	M	50 y	severe sunburn from 1 y	**intron 1 c.207+196T>A**	exon 11 c.2749T>C p.*917Rext*83	SCC (nose, 49 y), BCC (nose, 49 y)	6	this study
XP95NG	M	40 y	severe sunburn from infancy	**intron 1 c.207+196T>A**	exon 11 c.2749T>C p.*917Rext*83	no skin cancer	10	this study
XP18NG	F	12 y	no history of severe sunburn abnormal pigmented spots from 10 y	**intron 1 c.207+196T>A**	exon 11 c.2480_2481delCA p.T827Sfs*9	no skin cancer	33	this study
XP133KO	F	24 y	blister formation after sunbathing at 3 mo	**intron 1 c.207+196T>A**	exon 8 c.1470delGp.K491Sfs*2	no consanguinity, increase in pigmented maculae from school age childhood, congestion of conjunctiva upon sun exposure	16	this study
XP23OS	F	45 y	increase in pigmented maculae from 6 y	**intron 1 c.207+196T>A**	exon 8 c.1364dupAp.E456Gfs*8	No evidence of skin cancer	7	Ref. 38
XP96NG	F	71 y	severe sunburn from childhood	**intron 1 c.207+196T>A**	exon 4 c.767delAp.N256Mfs*29	SCC (lower lip, 71) decreased MED (25 mJ/cm^2^)	11	this study
XP2YO	F	64 y	extensive sunburn from 2 mo	**intron 8 c.1811+326C>T**	**intron 8 c.1811+326C>T**	tiny pigmented maculae on the sun-exposed area, SCC on lower lip (64), die of bile duct carcinoma (65 y)	4	Ref. 39
XP3YO	M	29 y	pigmented plaque on the sun-exposed area from early childhood	**intron 8 c.1811+326C>T**	**intron 8 c.1811+326C>T**	grandnephew of XP2YO, keratoacanthoma (26 y)	9	Ref. 39

### XP133KO.

A 24-y-old Japanese female was referred to Kobe University hospital. At the time of the referral, she had an increased number of freckle-like pigmented maculae on her face, bilateral upper extremities, and shoulders. At the age of 3 mo, she had experienced severe sunburn with blister formation; since then she had been photo-protected by her mother. At the age of 7 to 8 y, she noticed an appearance of freckle-like pigmented maculae on her face. She had conjunctival congestion when she got sunburnt. At the age of 22 to 23 y, she underwent CO_2_ laser therapy for seborrheic keratosis of her cheeks; she also consulted an increase in multiple pigmented maculae on her face, arms, and forearms. Since the age of 22 y, she had been working as a nursery teacher, spending much time outdoors. Her parents were not consanguineous. Her two siblings did not present pigmented maculae.

### XP165KO.

A 44-y-old Japanese male was referred to Kobe University Hospital. At the time of the referral, he had multiple pigmented maculae and he developed multiple basal cell carcinomas (BCCs) on his face. Since his early childhood, he had experienced severe sunburn with blister formation; the exaggerated sunburn had remained 1 to 2 wk. During his school years, he was a member of a football club; he had experienced severe sunburn with purplish erythema after playing football. Since then, he had protected himself from sun exposure in his daily life. At the age of 35 y, he noticed an appearance of pigmented maculae. At the age of 44 y, he consulted a dermatologist for black pigmented papules on the bilateral nose alae, left cheek, and right ante-auricular region, all of which were histologically diagnosed as BCCs. He had no history of developmental retardation or epilepsy. He did not manifest photophobia, apparent neurological abnormalities, or hearing impairment. Minimal erythema dose (MED) at 24 h after UV exposure was 60 mJ/cm^2^; however, it decreased to 30 mJ/cm^2^ at 48 and 72 h after UV exposure.

### XP136KO.

A 44-y-old Japanese female was referred to Kobe University Hospital. At the time of the referral, she developed 2BCCs on her face. At the age of 3 y, she had experienced severe sunburn and photophobia. MED decreased to 30 mJ/cm^2^; the peak of erythema was delayed.

### XP97NG.

A 71-y-old Japanese female was referred to Osaka Medical and Pharmaceutical University Hospital. At the time of the referral, she had severely photodamaged skin on sun-exposed areas. She had no skin cancer. At the age of 16 y, she had noticed gradually increasing freckle-like pigmented macules on the sun-exposed areas. As she was a farmer and she had noticed mild photosensitivity, she had a habit of sun protection by physical sunscreens and cosmetics. She had no neurological abnormalities. Her parents were not consanguineous. Her two siblings also developed similar skin abnormalities. She was assigned to XP-F by complementation test (plasmid host cell reactivation assay).

### XP4NG.

Original case report was published previously in Japanese (37). A 64-y-old Japanese male was referred to Nagasaki University Hospital. At the time of the referral, he developed actinic keratosis and seborrheic keratosis, although he manifested multiple pale brown pigmented maculae on sun-exposed areas, including face, neck, V area of the anterior chest, and arms. At the age of 5 y, he had noticed an increased number of pigmented maculae on sun-exposed areas. In his school age, he had often noticed erythema on sun-exposed areas. Since then, he had a habit of sun protection by wearing clothes with long sleeves and a hat. At the age of 61 y and thereafter, he had noticed photophobia and ophthalmalgia. He had no neurological abnormalities. MED decreased to 28 mJ/cm^2^; the erythema peak was delayed at 48 h after UV exposure.

During our practical XP diagnosis, we initially performed UDS (measures GG-NER activity) and RRS (recovery of RNA synthesis, measures TC-NER activity) tests of fibroblasts derived from the cases and determined their complementation groups (40). Representative data are shown (XP43NG: Fig. 1*B* and *SI Appendix*, Fig. S1 *A* and *B*; XP2YO: Fig. 1*C* and *SI Appendix*, Fig. S1 *C* and *D*). All cases described in this report were both UDS and RRS defective, indicating that they were compromised in both GG-NER and TC-NER pathways (*SI Appendix*, Table S1). Except for two cases without cellular materials, ectopic expression of the *ERCC4*/*XPF* cDNA by lentivirus infection exclusively rescued the UDS/RRS-deficiency in the patients’ cells, confirming that they are all assigned to the XP-F complementation group (Fig. 1 *B* and *C* and *SI Appendix*, Table S1).

### Deep-Intronic Variants Identified in the XP-F Cases.

**Fig. 2.**
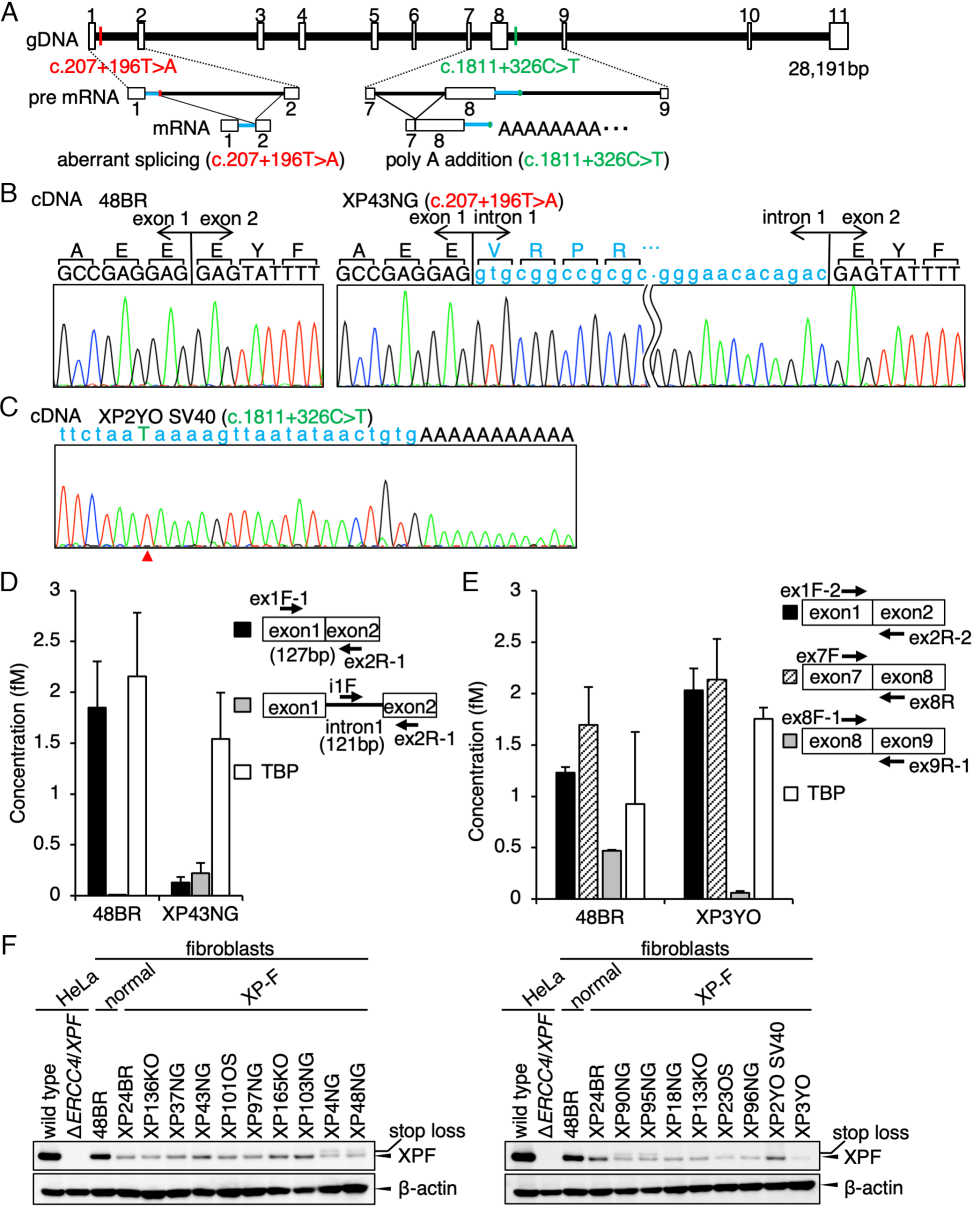
Locations of the *ERCC4*/*XPF* intron variants identified in the Japanese XP-F cases. (*A*) Estimated structures of pre-mRNA products resulting from the *ERCC4*/*XPF* intron variants. Cryptic intron fragments identified in the patients’ mRNA are shown in blue lines. (*B*) cDNA sequences of the *ERCC4*/*XPF* exons 1 to 2 boundary in 48BR (normal) and XP43NG (XP-F). The 5′ cryptic intron 1 fragment is shown in blue letters. (*C*) A cDNA sequence of the *ERCC4*/*XPF* exon8–intron9 boundary in XP2YO (XP-F). The cryptic intron 8 fragment is shown in blue letters. Arrowhead indicates the variant position. (*D*) Digital quantitative PCR (digital qPCR) detected the reduction of *ERCC4*/*XPF* expression and the aberrant splicing product of intron 1 in XP43NG (filled bars, PCR amplifying the *ERCC4*/*XPF* exons 1 to 2 boundary; gray bars, PCR amplifying the 5′ cryptic fragment of *ERCC4*/*XPF* intron 1; open bars, a control PCR product of the *TBP* gene). (*E*) Digital qPCR detected the reduction of *ERCC4*/*XPF* expression in XP3YO (XP-F) (filled bars, PCR amplifying the *ERCC4*/*XPF* exons 1 to 2 boundary; hatched bars, exons 7 to 8 boundary; gray bars, exons 8 to 9 boundary; open bars, *TBP*). (*F*) Immunoblotting of the XPF protein. wild type and Δ*ERCC4*/*XPF*, wild type and *ERCC4*/*XPF*-deficient HeLa cells; 48BR, normal; XP24BR, XP-F control; [XP136KO, XP37NG, XP43NG, XP101OS, XP97NG, XP165KO, XP103NG, XP4NG, XP48NG, XP90NG, XP95NG, XP18NG, XP133KO, XP23OS, XP96NG, XP2YOSV40, and XP3YO], the Japanese XP-F cases. b-actin (ACTB) as a loading control. XPF-upper bands represent the stop-loss product, p.*917Rext*83. The PCR primers are listed in *SI Appendix*, Fig. S2 and Table S2.

To determine disease causative mutations of the cases, we then performed Sanger sequencing of all the exons of *ERCC4*/*XPF* gene. Of 17 cases, eight patients carried at most one exonic *ERCC4*/*XPF* pathogenic/likely pathogenic mutations, and others had no obvious potentially pathogenic variants in *ERCC4*/*XPF* exons (Table 1). We therefore presumed that their unidentified pathogenic variants would be located in introns or regulatory regions of *ERCC4*/*XPF*. To identify additional pathogenic variants of the cases, we further conducted whole genome sequencing (WGS) and Sanger sequencing. Besides common variants, we identified either of two rare SNPs located in intron 1, c.207+196T>A [rs1175905917 (MAF: 33/16,760, 8.3K Japanese; 1/1,552, gnomAD East Asia), spliceAI score = 0.41 (Donor Gain)] (Fig. 2*A* red; *SI Appendix*, Fig. S2*A*), or in intron 8, c.1811+326C>T (not reported in public databases) (Fig. 2*A* green; *SI Appendix*, Fig. S2*B*), shared among all of the cases (Table 1).

To define the consequences of these deep intronic variants, we analyzed the *ERCC4*/*XPF* transcripts. We performed direct cDNA Sanger sequencing in the following representative cases, XP43NG bearing c.207+196T>A (intron 1 variant) and XP2YO carrying c.1811+326C>T (intron 8 variant). As shown in Fig. 2*A* (red) and Fig. 2*B*, the *ERCC4*/*XPF* cDNA derived from XP43NG contained an intronic segment of 192 bp between exons 1 and 2. This intronic fragment was derived from the exon1–intron1 boundary to the upstream of the c.207+196T>A variant, suggesting that this intron variant activates a cryptic 5′ donor splicing site and causes the aberrant mRNA product. In contrast, cDNA derived from XP2YO contained a partial intron 8 fragment with 3′ truncation of exons 9 to 11, indicating that an alternative polyadenylation occurred at the downstream of the c.1811+326C>T variant (Fig. 2*A* green, Fig. 2*C*).

To further study the effects of these intron variants in the stability of mRNA, we quantified the expression level of *ERCC4*/*XPF* in the patients’ cells by quantitative digital PCR (see *SI Appendix*, Table S2 for PCR primer sets). In XP43NG cells, a PCR primer set amplifying the *ERCC4*/*XPF* cDNA containing the exon1–exon2 boundary determined a significant reduction of *ERCC4*/*XPF* expression compared to wild type (Fig. 2*D* filled bars). The aberrant cDNA with the cryptic intron 1 fragment, which leads to lack ~90% of the XPF protein, was also detected only in XP43NG cells (Fig. 2*D* gray bars). These data indicate that the intron 1 variant causes the reduction of *ERCC4*/*XPF* expression due to nonsense-mediated mRNA decay (NMD). Similarly in XP3YO cells, the full-length *ERCC4*/*XPF* cDNA, amplified by a primer set that spans the *ERCC4*/*XPF* exon8–exon9 boundary, was also significantly reduced (Fig. 2*E* gray bars), although the mRNA with the partial intron 8 fragment looks substantially stable (Fig. 2*E* filled/hatched bars). As the truncation leads to abolishing the nuclease- and the ERCC1-binding domains, the expressed protein is inactive.

We then confirmed the significant reduction of the XPF protein expression in all cases by immunoblot analysis; in addition, the aberrant proteins derived from the intron variants were not detectable (Fig. 2*F*). Of note, four cases bearing an exonic stoploss variant, p.*917Rext*83 (the mutation results in an extra 83 amino acids after the wild-type final lysine residue; this form in unstable), showed a doublet of the XPF protein bands (Fig. 2*F*: XP4NG, XP48NG, XP90NG, and XP95NG). Collectively, in all cases, the mutant proteins are not expressed and/or unstable, or even expressed they are lacking the essential functional domains; therefore, they are naturally inactive. Nevertheless, we detected a small quantity of the normal intact form of the XPF protein in all patients. Since the ERCC1-XPF endonuclease is essential for cell survival and development, we presume that the patients are surviving as some of the correct mRNA splicing and polyadenylation still occur in the cells.

### SNP Analysis Reveals Common Haplotype Blocks Shared among the Patients.

To evaluate whether these intronic variants are founder mutations, we performed SNP analysis using the WGS data of the patients. Initially, we analyzed haplotype data in HapMap from Japanese samples (41) to determine linkage-disequilibrium (LD) blocks located near the *ERCC4*/*XPF* gene. We identified an LD block that spans ~55 kbp, including the entire *ERCC4*/*XPF* gene (*SI Appendix*, Fig. S2*C*). We also calculated LD-units (LDU) of the 1,000 Genomes Project (1KGP) haplotypes from Japanese population (42) and generated a cumulative LDU map for the identified region (*SI Appendix*, Fig. S2*D*). These LD analyses indicate that the *ERCC4*/*XPF* intronic variants both coincide with the LD block, suggesting that the variants segregate nonrandom manner.

To determine common haplotypes shared among the patients, we then extracted tag-SNP haplotypes within the ~55 kbp LD region from the patients (WGS) as well as from HapMap Japanese/Chinese individuals (Table 2). Within 5 haplotype combinations (Haplotype blocks1~5), the intron 1 variant (see rs1175905917, highlighted in red) segregates only with Haplotype block1 in homozygous patients. Cosegregation of the intron 1 variant with Haplotype block1 was also confirmed for a representative heterozygous patient, XP133KO, by long-read sequencing. Similarly, the intron 8 variant (see position chr16:14029926, highlighted in green) cosegregates with Haplotype block5. Collectively, these LD/haplotype analyses suggest that the identified intronic variants are founder mutations inherited from common ancestral origins.

**Table 2.** Common haplotype blocks identified in the XP-F patients

SNP ID	rs3888364	rs753191	rs12597883	rs1175905917	-	rs8060520	Haplotype frequency	
Position (chr16)	13996140	14000581	14007563	14014425	14029926	14051776		
Ref/Alt	A/G	T/A	G/A	T/A	C/T	C/G		
MAF	0.650	0.374	0.221	0.002	0	0.311	JPT	CHB
Haplotype 1	G	A	A	T	C	C	0.119	0.060
Haplotype 2	**A**	**T**	**G**	T	C	C	0.190	0.185
Haplotype 3	G	**T**	**G**	T	C	**G**	-	-
Haplotype 4	G	A	**G**	T	C	C	0.120	0.162
Haplotype 5	G	A	A	T	C	**G**	-	0.056
Sample ID							Haplotype combination	
**XP136KO**	G	A	A	**A**	C	C	1	Allele1
	G	A	A	**A**	C	C	1	Allele2
**XP37NG**	G	A	A	**A**	C	C	1	
	G	A	A	**A**	C	C	1	
**XP101OS**	G	A	A	**A**	C	C	1	
	G	A	A	**A**	C	C	1	
**XP97NG**	G	A	A	**A**	C	C	1	
	G	A	A	**A**	C	C	1	
**XP165KO**	G	A	A	**A**	C	C	1	
	G	A	A	**A**	C	C	1	
**XP103NG**	G	A	A	**A**	C	C	1	
	G	A	A	**A**	C	C	1	
**XP43NG**	G	A	A	**A**	C	C	1	
	G	A	A	**A**	C	C	1	
**XP4NG**	G	A	A	**A**	C	C	1	
	**A**	**T**	**G**	T	C	C	2	
**XP48NG**	G	A	A	**A**	C	C	1	
	**A**	**T**	**G**	T	C	C	2	
**XP133KO**	G	A	A	**A**	C	C	1	
	**A**	**T**	**G**	T	C	C	2	
**XP23OS**	G	A	A	**A**	C	C	1	
	**A**	**T**	**G**	T	C	C	2	
**XP90NG**	G	A	A	**A**	C	C	1	
	**A**	**T**	**G**	T	C	C	2	
**XP95NG**	G	A	A	**A**	C	C	1	
	**A**	**T**	**G**	T	C	C	2	
**XP18NG**	G	A	A	**A**	C	C	1	
	G	**T**	**G**	T	C	**G**	3	
**XP96NG**	G	A	A	**A**	C	C	1	
	G	A	**G**	T	C	C	4	
**XP2YO**	G	A	A	T	**T**	**G**	5	
	G	A	A	T	**T**	**G**	5	
**XP3YO**	G	A	A	T	**T**	**G**	5	
	G	A	A	T	**T**	**G**	5	

### Minigene Assay: Aberrant mRNA Splicing and Alternative Polyadenylation Are the Causes of Nonsense-Mediated mRNA Decay.

To further study that the identified intron variants exclusively cause the aberrant mRNA processing, we performed minigene splicing assay. We had constructed GFP-*ERCC4*/*XPF* partial gene fusion vectors with the identified intron variants.

**Fig. 3.**
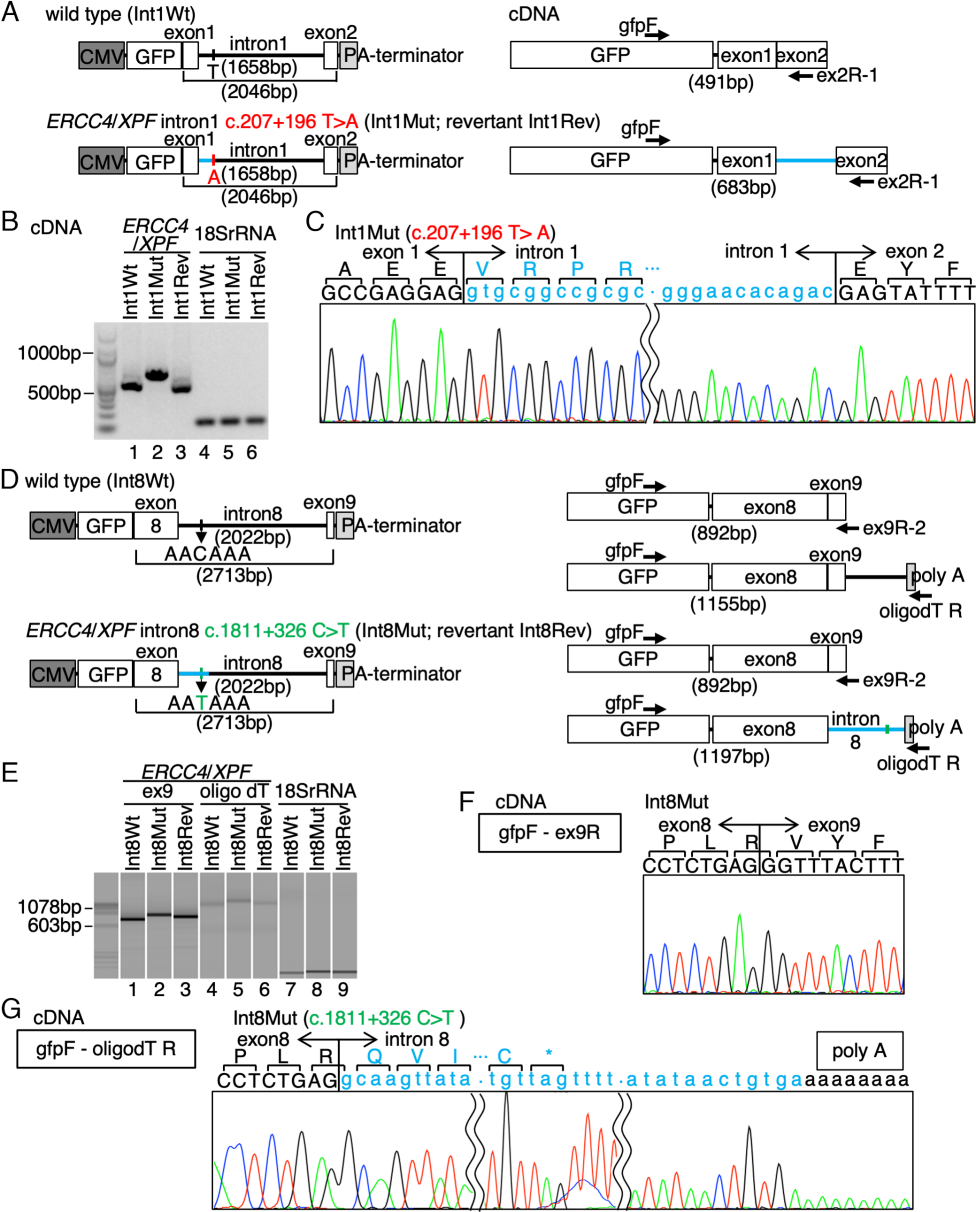
Aberrant pre-mRNA splicing and alternative polyadenylation events of *ERCC4*/*XPF* in the cases. (*A*) Schematic representation of the minigene assay. GFP-*ERCC4*/*XPF* partial gene fusion vectors with or without the intron 1 variant, their corresponding transcripts, and the size of PCR amplified cDNAs are shown. Int1Wt, a wild-type vector with the wild-type intron 1 sequence amplified from normal 48BR cells; Int1Mut, a mutant vector with the intron 1 variant amplified from XP43NG; Int1Rev, a vector with a wild-type revertant of the intron 1 variant generated from Int1Mut. (*B*) The GFP-*ERCC4*/*XPF* minigene cDNA products were resolved on agarose-gel electrophoresis. RT-PCR was performed using the primers shown in *A*. Int1Wt and Int1Rev gave the same sized band, while Int1Mut gave a 192bp longer product. 18SrRNA as a control. (*C*) Direct Sanger sequencing of the cDNA products. Int1Mut gave a fragment with the cryptic intron 1 insertion. (*D*) Minigene vectors with or without the intron 8 variant and the corresponding transcripts are shown. Int8Wt, a wild-type vector with the wild-type intron 8 sequence; Int8Mut, a mutant vector with the intron 8 variant amplified from XP3YO; Int8Rev, a wild-type revertant of the intron 8 variant generated from Int8Mut. (*E*) Minigene cDNA products were resolved on a capillary-gel electrophoresis system, MultiNa (SHIMADZU). ex9 denotes the PCR products that spans exons 8 to 9, being amplified from the normal transcript, while oligodT represents the PCR products amplified from both normal- and 3′ truncated-mRNAs. (*F* and *G*) Direct Sanger cDNA sequencing of the exons 8 to 9 boundary (*F*) and the transcription termination site (*G*). Int8Mut gave a fragment with the cryptic intron 8 insertion. The PCR primers are listed in *SI Appendix*, Fig. S3 and Table S2.

The *ERCC4*/*XPF* genomic DNA fragments (exon1–intron1–exon2) were PCR amplified from wild-type 48BR and XP43NG cells; they were cloned into the downstream of a GFP coding cDNA (Fig. 3*A*). Both wild-type (Int1Wt) and the intron 1 variant, c.207+196T>A (Int1Mut), loci were examined by Sanger sequencing, and the GFP-fusion transcripts were collected from wild type HeLa cells transfected with the plasmids. cDNA fragment length analysis (Fig. 3*B*, lanes 1 to 2) and direct cDNA Sanger sequencing (Fig. 3*C* and *SI Appendix*, Fig. S3*A*) confirmed that the aberrant splicing product with the additional 192bp partial intron 1 fragment was only detected from cells transfected with the mutant Int1Mut plasmid. Moreover, transcripts from cells with a wild-type revertant plasmid (Int1Rev), which was generated from the Int1Mut vector by site directed mutagenesis of the variant locus, confirmed that c.207+196T>A was solely responsible for the intron 1 aberrant splicing event (Fig. 3*B*, lane 3, and *SI Appendix*, Fig. S3*A*).

Similarly, to test that the intron 8 variant, c.1811+326C>T, contributes to the cryptic polyadenylation event, the *ERCC4*/*XPF* exon8–intron8–exon9 genomic fragments were amplified from wild-type and XP3YO cells, and they were cloned into the GFP vector to generate Int8Wt and Int8Mut plasmids, respectively (Fig. 3*D*). Int8Rev, a revertant for the intron 8 variant, was also generated from Int8Mut. After transfection of the plasmids, transcripts were analyzed as above described. Although we noticed that the fragment size difference was not remarkable (Fig. 3*E*) and that the normal transcript was also detectable (Fig. 3*F* and *SI Appendix*, Fig. S3*B*), the premature transcription termination and cryptic polyadenylation events were only observed from the mutant transcripts (Fig. 3*G* and *SI Appendix*, Fig. S3*C*). Int8Rev transcripts also confirmed that c.1811+326C>T was solely responsible for the intron 8 aberrant polyadenylation event.

Collectively, these data confirmed that these rare intron variants were exclusively pathogenic and no other variants in introns 1 and 8 were not the cause of XP.

### Treatments with Antisense Oligonucleotides Designed for the Intronic Mutations Rescued Cellular DNA-Repair Defects.

**Fig. 4.**
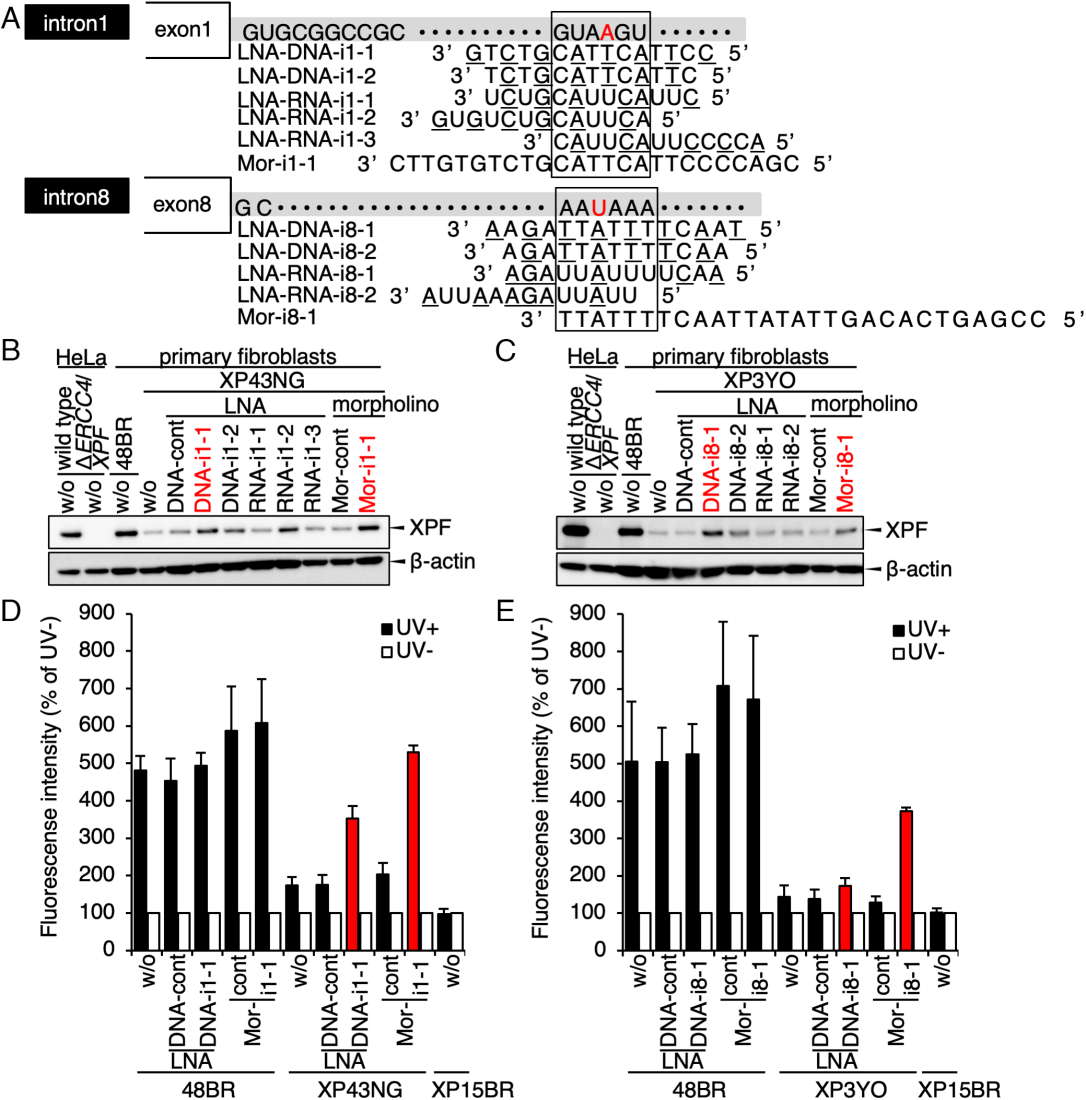
Recovery of DNA repair activity by antisense nucleotide treatments. (*A*) Schematic representation of the artificial antisense oligonucleotides (ASOs) designed for the intron 1 and the intron 8 variants. (*B* and *C*) Recovery of the XPF protein expression by the ASO treatments. (*B*) XP43NG, bearing the intron 1 variant, was treated with the LNA- as well as with the morpholino-modified oligonucleotides shown in *A*. (*C*) XP3YO, with the intron 8 variant, was treated with the ASOs shown in *A*. (*D* and *E*) Recovery of DNA repair activity by the ASO treatments. The reduced UDS was restored by the ASOs designed for the intron 1 variant in XP43NG cells (*D*) and for the intron 8 variant in XP3YO cells (*E*) (filled bars, 20 J/m^2^ UVC; open bars, without UV). Red bars indicate appropriate recoveries of *ERCC4*/*XPF* expression and DNA repair activity by the ASO treatments. The ASOs are listed in *SI Appendix*, Fig. S4 and Tables S3 and S4.

Artificial antisense oligonucleotides (ASOs) can be used to correct abnormal splicing events; therefore, they have a potential of treating various diseases (43). To evaluate that the identified XP intron mutations would be potential therapeutic targets, we designed ASOs that can interfere with the 5′ cryptic donor splice site as well as the alternative polyadenylation site, both created by the pathogenic intron mutations (scheme, Fig. 4*A*; sequences, *SI Appendix*, Tables S3 and S4). LNA- (locked nucleic acids, both DNA and RNA) and morpholino-oligos were chosen because of their efficiency and lower cytotoxicity, respectively (44, 45). The origin and design details of the ASOs as well as their off-target profiles, calculated by GGGenome, an ultrafast sequence search tool (DBCLS, Japan), are described in *Materials and Methods* and in Dataset S1 (summarized in *SI Appendix*, Table S5).

We first tested the correction of *ERCC4*/*XPF* pre-mRNA splicing event of the intron 1 variant site in XP43NG cells by the designed ASOs (Fig. 4*B*). After treatment with ASOs, the XPF protein expression was recovered to the normal level, indicating that the mRNA expression was efficiently restored by the ASOs. The efficiency was also confirmed in other XP-F cell lines with the intron 1 variant (*SI Appendix*, Fig. S4*A*). Similarly, the XPF protein expression was rescued by other ASOs designed for the intron 8 variant in XP3YO cells (Fig. 4*C*), indicating that the alternative polyadenylation event was also successfully inhibited and the *ERCC4*/*XPF* mRNA expression was restored by the ASO treatments. Note that all these ASOs and control oligonucleotides had no deleterious cellular toxicity affecting the XPF protein expression in wild-type cells (*SI Appendix*, Fig. S4*B*); however, the efficiency of recovery varied among the ASOs (Fig. 4 *B* and *C* and *SI Appendix*, Fig. S4*A*). This can be explained from the specificity of ASOs rather than their base chemical compounds, as we noticed that ASOs with less off-targets in genic regions gave better recovery (*SI Appendix*, Table S5); therefore, we decided to use selective ASOs (shown in red) for further experiments. Recovery of the *ERCC4*/*XPF* mRNA expression in consequence of inhibition of the aberrant splicing and the alternative polyadenylation events were also confirmed by digital qPCR (*SI Appendix*, Fig. S4 *C* and *D*).

To evaluate that the ASOs can also rescue the cellular XPF function, we assessed DNA repair capacity of the patient cells after treatments. Although the control and targeting ASOs have no effect on wild-type cells, the LNA- and morpholino-based ASOs designed for the intron 1 variant significantly restored the UDS activity of XP43NG cells (Fig. 4*D*). Similarly, only the morpholino oligonucleotide designed for the intron 8 variant also corrected the UDS defect of XP3YO cells (Fig. 4*E*). Correctively, these data indicate that the DNA repair defect of the patients’ cells was restored by the ASO treatments, suggesting that these intron variants can be good therapeutic targets for the XP-F cases (see schematic representations in *SI Appendix*, Fig. S4 *E* and *F*).

## Discussion

Among XP complementation groups, XP-F is a rare form, frequency of which in the worldwide population is ~1% of the entire XP cases (5, 7). By contrast, in Japan, XP-F is the fourth most frequent (4 to 7%) complementation group; therefore, most of the reported XP-F cases are from the Japanese population (3, 5). To date, all previously published *ERCC4*/*XPF* pathogenic variants have been identified in the exons, and with a limited number of variants resulting in devastating XFE and COFS, most of the pathogenic mutations are hypomorphic changes causing only mild XP clinical manifestations (31). In an extreme case, a hypomorphic variant associated with XP-F, p.P379S, is highly prevalent (rs1799802, MAF = 0.0041 in GnomAD); XP patients having this mutation only display very mild skin phenotypes due to residual endonuclease activity (46, 47). This is because vast majority of photosensitive individuals carrying this mutation may not be recognized as having XP (47, 48). It is therefore assumed that XP-F cases would be underdiagnosed and that XP-F patients with undefined pathogenic changes would have mutations in the introns as well. In this report, we, for the first time, described two *ERCC4*/*XPF* deep intronic rare variants responsible for 17 Japanese XP-F cases. All the cases are typical XP, showing skin phenotypes and early-onset skin tumors without marked neurological abnormalities. Importantly, the prevalence of the intron 1 variant, c.207+196T>A, is considerably high (MAF = 1/508) in the Japanese population, accounting for ~10% of the Japanese XP cases. According to the Hardy–Weinberg formula, the frequency of homozygotes for the intron 1 variant is expected to be 3.8 × 10^−6^ (0.00196 ^ 2). Based on the Japanese population (~126 million), this estimate suggests that there would be ~480 individuals who are homozygous for this variant. Despite this, most carriers of this variant, similar to the *XPF/ERCC4* p.P379S, may not be diagnosed with XP because of their relatively mild clinical phenotypes. Alternatively, many patients with this intron variant may remain undiagnosed because of the difficulty of genetic testing. At all events, XP-F now comes the third most-frequent complementation group in the Japanese population only to XP-A (53%) and XP-V (31%). This intron 1 variant should be included in routine clinical genetic testing of Japanese XP cases by Sanger sequencing.

Both *ERCC4*/*XPF* intron variants identified caused a reduction of *ERCC4*/*XPF* expression due to NMD (49). This may partly explain the relatively mild XP-F manifestations of the cases because of the normally functioning residual XPF protein expression. The transcribed intron 1 variant activates the 5′cryptic splicing donor site, which is predicted to have a higher binding affinity of U1snRNA than the original site (*SI Appendix*, Fig. S4*E*). Similarly, the intron 8 variant results in the polyadenylation consensus sequence (5′-AAUAAA-3′), which is a target for cleavage and polyadenylation specificity factor(*SI Appendix*, Fig. S4*F*). Both mutations interfere with the normal *ERCC4*/*XPF* mRNA processing, which eventually induces the aberrant pathogenic mRNA production and NMD.

### A Long-Standing Problem with the Use of XP2YO/XP3YO Materials.

XP2YO- and XP3YO-derived cell lines have been extensively used for decades in molecular and cellular biological studies in the NER field. These cell lines initially contributed to defining the functionality of the human ERCC1-XPF endonuclease complex and the defective gene (*ERCC4/XPF*) in XP-F patients (50, 51). They have been fundamental materials as *ERCC4*/*XPF*-defective cell lines used for various genetic complementation tests of XP cases as well as for the sources of recipient cellular extracts used in in vitro NER reconstitution assays (25). NER researchers invested substantial efforts into searching for pathogenic variants in these cell lines; however, no one has conclusively pointed out the causative mutations. Within the studies, Takebe (Kyoto University) and his colleagues reported that XP2YO and XP3YO had carried four different exonic variants in the cDNAs (XP2YO: p.T567A, p.V657Efs*28; XP3YO: p.R490Q, p.L608P) (52). On the other hand, Richard D. Wood (MD Anderson Cancer Center) and his colleagues had conducted sequencing analysis and initially noticed the absence of any potentially pathogenic variants within the cDNAs or the promoter region of genomic DNA from these cell lines. In this study, we confirmed that none of the exonic variants were present. We assume the discrepancy is due to an erroneous Sanger sequencing of the cloned *ERCC4/XPF* cDNAs performed in the Matsumura et al. paper. Our findings finally solve the outstanding issue in the NER field by locating the pathogenic variant in widely used, historically important XP-F cell lines.

### XP Therapeutics by Antisense Oligonucleotides.

Engineered nucleic acids such as artificial antisense oligonucleotides (ASOs) have a potential of treating various diseases. In recent years, ASO drugs have been approved for the treatment of several genetic disorders, such as Duchenne muscular dystrophy and spinal muscular atrophy (43). ASOs designed for certain pathological intron sequences can bind to disease-causing pre-mRNAs and interfere with alternative mRNA processing events (53). It increases the target mRNA stability; therefore, such ASO treatments can mitigate clinical features by resuming normal gene expression. This is because pathogenic intron variants can be possible therapeutic targets. We demonstrated that the reduced expression of *ERCC4*/*XPF* and the DNA repair deficiency can be restored by the treatment with ASOs.

Pharmacological treatment of systemic genetic disorders is generally difficult. Particularly, gene therapy requires a safe delivery of expression vectors to all affected organs; therefore, there is no established treatment for XP with neurologic symptoms. Since all of the XP cases reported here only display cutaneous manifestations, we would consider to attempt a pharmaceutical intervention by developing topical drug with anti-XP medical properties. A past study developed a skin lotion with liposomes containing a DNA repair enzyme, *Escherichia coli* T4 endonuclease V (54). This approach aimed to compensate for the compromised NER activity by removing UV-induced DNA lesions with the topically applied enzyme. In the trial, the treatment lowered the rate of development of actinic keratoses and BCCs in XP patients. Prospect of ASO-based therapeutics for genodermatoses is also supported by a clinical trial (Phase I/II) of QR-313 for dystrophic epidermolysis bullosa due to mutations of the *COL7A1* gene (55). As our study demonstrated that ASOs designed for intron pathogenic variants successfully restored DNA repair activity in XP-F patients cells, ASO-based treatment can be a potentially effective therapeutic approach for XP.

## Materials and Methods

### Human Studies.

The full study protocol was reviewed and approved by the following institutional review boards: medical ethics committees at Kobe University (approval number 160); medical ethics committees at Osaka Medical and Pharmaceutical University (approval number 2569-1); the Nagasaki University Ethical, Legal and Social Implications Committee; medical ethics committees at Kumamoto University (approval number 232); the Ethics Committee for Human Genome Studies in Research Institute of Environmental Medicine, Nagoya University (approval number 337). XP patients and healthy control samples were obtained with written informed consent. We only publish the patients’ haplotype data near the pathogenic loci.

### Cell Culture.

The following human cell lines were used in this study: 48BR, normal primary fibroblast; XP15BR, primary fibroblasts from an XP-A patient; XP24BR, an XP-F patient of a control; XP136KO, XP37NG, XP43NG, XP101OS, XP97NG, XP165KO, XP103NG, XP4NG, XP48NG, XP90NG, XP95NG, XP18NG, XP133KO, XP23OS, XP96NG, and XP3YO, primary fibroblasts from the described XP-F patients; XP2YOSV40, SV40 immortalized fibroblasts from the XP-F patient; HeLa, human cervical cancer cell line (wild type and the *ERCC4*/*XPF* gene deficient). All cells were maintained in Dulbecco’s modified Eagle medium (DMEM) (FUJIFILM Wako Pure Chemical Corporation, WAKO) supplemented with 10% fetal bovine serum (FBS) (Thermo Fisher Scientific) and 1% Penicillin-Streptomycin (WAKO), unless otherwise noted.

### UDS and RRS Assays.

Detailed protocols are described previously (40). Cells were cultured in DMEM (10% FBS) and plated in 96-well plastic plates (Corning). Cells were then UVC (254 nm) irradiated (20 J/m^2^ for UDS and 12 J/m^2^ for RRS assays). UDS was measured by the fluorescence-based 5-ethynyl deoxyuridine (EdU)-incorporation assay (56). Similarly, RRS levels were measured by the 5-ethynyl uridine (EU)-incorporation after 12-h incubation for RNA synthesis recovery (22, 23, 57). Nuclear fluorescent intensity was measured by high contents imaging systems (Thermo Fisher Scientific).

### Lentivirus Complementation.

Detailed protocols are described previously (40). The wild-type human *XPA* to *XPG* and *ERCC1* cDNAs were cloned into the pLenti6.3 viral vector (23). For the lentivirus production, HEK293 cells were transfected with the plasmids together with ViraPower Packaging Mix (Thermo Fisher Scientific) using Lipofectamine 2000 (Thermo Fisher Scientific). Forty-eight hours after transfection, lentivirus particles were purified and concentrated by PEG-it Virus Precipitation Solution (System Biosciences). For the complementation assays, the viruses were infected into XP43NG and XP2YOSV40 cells, as well as control XP15BR cells before UDS measurements.

### Immunoblotting.

Whole-cell lysates were prepared from the patient fibroblasts samples and resolved by 5 to 20% gradient-SDS-Poly-Acrylamide Gel Electrophoresis (PAGE) gels (WAKO). The resolved protein samples were then transferred to PVDF membranes (PALL Corporation), followed by blocking for 1 h at room temperature in 10% skim milk in TBST (25 mM Tris HCl pH 8.0, 135 mM NaCl, 0.05% Tween 20). The membranes were incubated with primary antibodies (mouse monoclonal anti-XPF, Ab-1, Thermo Fisher Scientific, 1:500; mouse monoclonal anti-b-actin (ACTB), sc-47778, Santa Cruz, 1:1000) in 5% skim milk in TBST (0.05% Tween 20) for 18 h at 4 °C. The membranes were washed three times in TBST (0.05% Tween 20), followed by incubation with HRP-conjugated secondary antibodies (Cell Signaling Technologies: anti-mouse, 7076, 1:1000) in 5% skim milk in TBST (0.05% Tween 20). After washing twice with TBST (0.05% Tween 20), the proteins were detected using Western Lightning Plus-ECL (PerkinElmer) with ImageQuant LAS 4000 mini (GE Healthcare). b-actin is a loading control.

### Genome Analysis.

Genomic DNA (gDNA) of the patients was fragmented by Picoruptor 2 (Diagenode). The gDNA fragments were sequenced on the Illumina HiSeq 2500 (Illumina) or the MGI DNB-SEQ T7 (MGI Tech Co., Ltd.) sequencers using paired-end (PE) flow cells to obtain 150–base pair PE reads of 30×coverage. The sequence data were analyzed by our standard pipeline (58). The reads were aligned to the human reference genome (GRC h37/hg19).

### SNP Analysis.

#### LD block extraction and visualization.

HapMap data from Japanese (JPT) and Chinese (CHB) individuals used in the SNP analyses were downloaded from the following websites:

https://ftp.ncbi.nih.gov/hapmap/genotypes/latest_phaseII+III_ncbi_b36/forward/non-redundant/genotypes_chr16_JPT_r27_nr.b36_fwd.txt.gz (JPT)

https://ftp.ncbi.nih.gov/hapmap/genotypes/latest_phaseII+III_ncbi_b36/forward/non-redundant/genotypes_chr16_CHB_r27_nr.b36_fwd.txt.gz (CHB)

The hg18 HapMap data were converted to BED format, followed by liftovered to hg19 genomic coordinates. Using the converted HapMap data, LD blocks, located within ~1 Mbp region (chr16:13875363-14989844), around the *ERCC4*/*XPF* gene were extracted. LD-heatmaps for the ~1 Mbp- and ~55 kbp (chr16: 13996140-14051776)- regions were visualized by Haploview software (59).

#### LDU calculation and plotting.

The 1000 Genomes Project (1KGP) data were downloaded from the following websites:

https://bochet.gcc.biostat.washington.edu/beagle/1000_Genomes_phase3_v5a/b37.vcf/chr16.1kg.phase3.v5a.vcf.gz (vcf file: haplotype data)

http://ftp.1000genomes.ebi.ac.uk/vol1/ftp/release/20130502/integrated_call_samples_v3.20130502.ALL.panel (reference panel: cohort information)

Haplotype data of the 1 KGP Japanese population were extracted from the vcf file using cohort information in the reference panel. Linkage Disequilibrium Units (LDU) were calculated and plotted against ~211 kbp (chr16:13,900,333-14,111,429) region using LDMAP (parameters for the calculation: minor allele frequency cutoff = 0.05; Hardy–Weinberg equilibrium deviation *P* value cutoff = 0.001) (60).

#### Long-read sequencing.

XP133KO patient’s gDNA was fragmented by g-TUBE (Covaris). Long-read sequencing libraries were prepared using PacBio SMRT bell prep Kit 3.0 (PacBio). Sequence data were obtained on the PacBio Sequel II sequencer, using a SMRT Cell 8M and Sequel II Sequencing Plate 2.0 reagents (movie time 30 h). Output HiFi (highly accurate reads) FASTQ were analyzed using SMRT Tools software (PacBio, alignment to hg19 human reference genome with pbmm2).

#### Digital qPCR.

Direct-zol RNA MiniPrep kit (ZYMO RESEARCH) was used to extract total RNA. The first strand was synthesized with Super Script IV reverse transcriptase (Thermo Fisher Scientific) with oligo dT primers. Digital quantitative PCR (digital qPCR) was performed using Eva Green ddPCR Supermix (Bio-Rad) and QX100 Droplet Digital PCR System (Bio-Rad). PCR conditions were as follows (95 °C 5 min; 95 °C 30 s, 60 °C 1 min, 40 cycles). PCR primers used for amplifying the designated *ERCC4*/*XPF* and *TBP* cDNA fragments are displayed in *SI Appendix*, Table S2.

#### Mini-Gene Constructs and In Vivo Splicing Assays.

The *ERCC4*/*XPF* intron 1 gDNA fragments were PCR amplified from 48BR (normal) and XP43NG (XP-F) cells and cloned into pAcGFP1-C3 vector (Clontech). The resultant constructs were designated as Int1Wt (48BR) and Int1Mut (XP43NG). Sanger sequencing was performed to confirm the vector-insert junction sequences as well as genomic SNPs and the pathogenic variant. A wild-type revertant, Int1Rev (A to T reversion at c.207+196), was generated from Int1Mut construct by site-directed PCR mutagenesis. Similarly, the *ERCC4*/*XPF* intron 8 gDNA fragments were also amplified from 48BR and XP3YO (XP-F) cells and cloned into the vector. The resultant constructs were designated as Int8Wt (48BR) and Int8Mut (XP3YO). A revertant (T to C reversion at c.1811+326) was generated from Int8Mut construct (Int8Rev). We were aware that Int8Wt and Int8Mut constructs contained PCR errors (e.g., differences in the number of homopolymer bases), but we confirmed that Int8Rev had an identical sequence to that of Int8Mut except the C-base located at the pathogenic variant locus. The constructed GFP plasmids were introduced in the wild-type HeLa cells using Lipofectamin 2000 (Thermo Fisher Scientific), followed by RNA extraction 24 h after transfection. 1st-strands were synthesized from total RNA samples using Super Script IV reverse transcriptase (Thermo Fisher Scientific). Designated PCR fragments were amplified with sets of primers (*SI Appendix*, Table S2) and analyzed by gel electrophoresis and Sanger sequencing.

#### Design of the Antisense Oligo Nucleotides.

Sequences of the antisense oligonucleotides (ASOs) are provided in *SI Appendix*, Tables S3 and S4. LNA (locked nucleic acids)-modified ASOs, made of DNA or RNA, were purchased from Ajinomoto Bio-Pharma Services (https://www.ajibio-pharma.com/). Morpholino ASOs were purchased from GENE TOOLS (https://www.gene-tools.com/). LNA-ASOs were designed based on the following principal criteria from a reference (45): 13 to 15 bp in total length; LNA-modifications (DNA or RNA) were introduced every other nucleotide if possible; in RNA-oligos, the modifications were introduced in other than uridine nucleotides. Morpholino ASOs were designed by the manufacturer based on the target sequences (strategies are not disclosed). Off-target profiles of the ASOs were calculated by GGGenome with the following criteria (https://gggenome.dbcls.jp/en/hg19/): database, human genome GRCh37/hg19; max number of mismatches/gaps, 0; search for both strands (Dataset S1). The off-targets in genic regions were summarized in *SI Appendix*, Table S5.

#### Transfection of ASOs.

LNA-ASOs were transfected using Lipofectamine 2000 (ThermoFisher) under the manufacturer’s instructions. Briefly, cells were seeded in plastic 6-96 well plates and cultured in DMEM (10% FBS, without antibiotics) for overnight. Transfection reagents were prepared in OptiMEM (ThermoFisher) mixed with Lipofectamine 2000 (1:100 dilution) and LNA-ASOs (final concentration 40 nM), followed by dilution in 1ml DMEM (10% FBS, without antibiotics). The culture media were replaced by the transfection reagents mixture. Four hours after the treatments, the media were replaced with fresh DMEM (10% FBS, with antibiotics). Cellular assays were conducted 24 h transfection. Morpholino-ASOs were transfected using Endo-porter (GENE TOOLS) under the manufacturer’s instructions. Similar to the LNA-ASOs transfection, transfection reagents were prepared in 1ml DMEM (10% FBS, without antibiotics) mixed with Endo-porter solution (final concentration six microM) and morpholino-ASOs (final concentration three microM). Cellular assays were conducted 24 h transfection without replacing the transfection media.

## Supplementary Material

Appendix 01 (PDF)

Dataset S01 (XLSX)

## Data Availability

All data needed to evaluate the conclusions are presented in the paper. Materials related to this paper are available from the corresponding author, Tomoo Ogi (togi@riem.nagoya-u.ac.jp), Department of Genetics, Research Institute of Environmental Medicine (RIeM), Nagoya University, Nagoya, Japan. All study data are included in the article and/or supporting information.
