# Supplementary material for: Deep intronic founder mutations identified in the ERCC4/XPF gene are potential therapeutic targets for a high-frequency form of xeroderma pigmentosum
Source: Proc Natl Acad Sci U S A. 2023 Jun 26;120(27):e2217423120. doi: 10.1073/pnas.2217423120 (PMC10318981; doi:10.1073/pnas.2217423120)
Supplement: Supplementary file 1 — Appendix 01 (PDF) [file pnas.2217423120.sapp.pdf]

PNAS

Supporting Information for
Deep intronic founder mutations identified in the *ERCC4*/*XPF* gene are potential therapeutic targets for a high-frequency form of xeroderma pigmentosum

Chikako Senju, Yuka Nakazawa, Taichi Oso, Mayuko Shimada, Kana Kato, Michiko Matsuse, Mariko Tsujimoto, Taro Masaki, Yasushi Miyazaki, Satoshi Fukushima, Satoshi Tateishi, Atsushi Utani, Hiroyuki Murota, Katsumi Tanaka, Norisato Mitsutake, Shinichi Moriwaki, Chikako Nishigori, Tomoo Ogi*

*Correpondence to Tomoo Ogi
Email: togi@riem.nagoya-u.ac.jp

This PDF file includes:

Figures S1 to S4
Tables S1 to S5

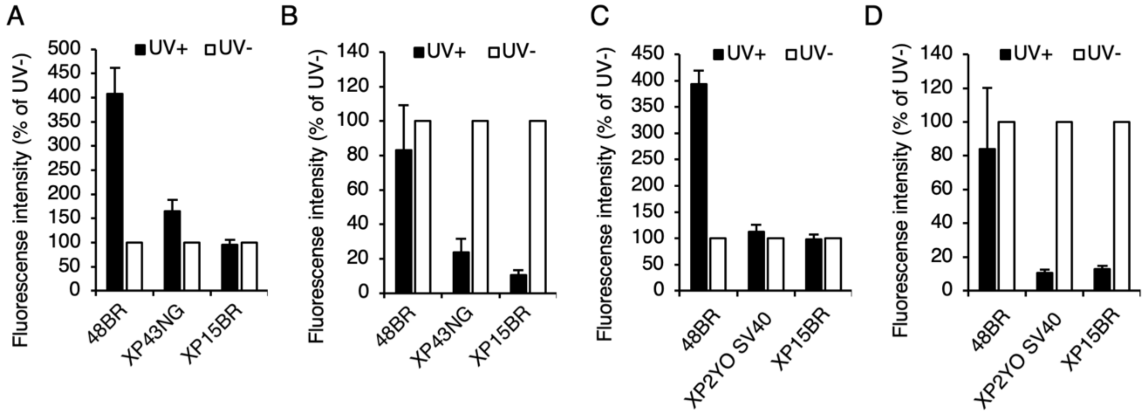

Fig. S1. NER deficiency in the Japanese XP-F cases

(**A**) Deficiency of UDS after UV-irradiation (filled bars, 20 J/m^2 UVC; open bars, without UV). UDS was measured by ethynyldeoxyuridine (EdU) incorporation and nuclear fluorescence measurement (UDS-assay). 48BR, normal; XP43NG, representative XP-F case-1; XP15BR, XP-A. (**B**) Deficiency of RNA synthesis recovery (RRS) after UV-irradiation (filled bars, 12 J/m^2 UVC; open bars, without UV). RRS was measured by ethynyluridine (EU) incorporation (RRS-assay). (**C**) Deficiency of UDS after UV-irradiation. XP2YOSV40 (SV40-immortalised), representative XP-F case-2. (**D**) Deficiency of RRS after UV-irradiation. UDS and RRS were normalised to activity in non-irradiated cells. Error bars: S.D. of means of triplicate experiments.

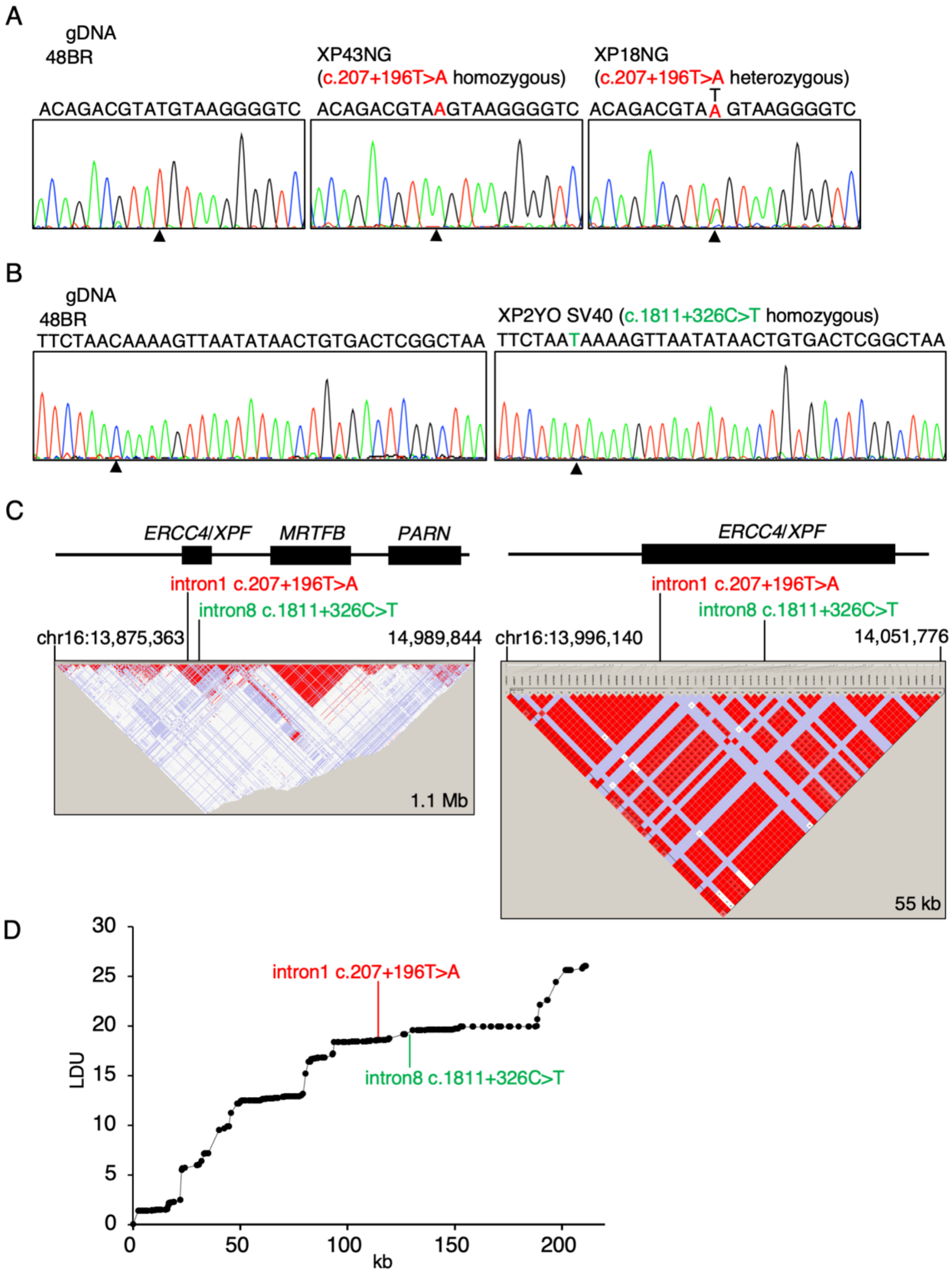

Fig. S2. The *ERCC4/XPF* intron variants in the Japanese XP-F cases are founder mutations (**A**) gDNA sequences of the intron 1 variant, c.207+196T>A (**red**), in representative XP-F cases, XP43NG (homozygous) and XP18NG (heterozygous). Arrowheads indicate the variant position. (**B**) gDNA sequences of the *ERCC4/XPF* intron 8 variant, c.1811+326C>T (**green**), in a representative XP-F case, XP2YO (homozygous). 48BR is a control (normal). (**C**) Linkage Disequilibrium (LD) heat-map of the HapMap Japanese population. Colored tiles with numbers represent Haploview LD (100×D', D'≠1) and their LOD (logarithmic odds) scores between tag-SNPs (standard color scheme: regions of high LD are colored in red, D'=1 & LOD>=2; low LD, D'<1 & LOD>=2, pink; low LOD, D'=1 & LOD<2, blue; no LD, white, D'<1 & LOD<2). Within ~1.1 M base pairs (chr16:13,875,363-14,989,844) (left panel) near the *ERCC4/XPF* gene are

presented. Both intron variants are located in the LD block (chr16:13,996,140-14,051,776) that spans ~55 kb, including the entire *ERCC4*/*XPF* gene (right panel). (**D**) LD units (LDU) of the 1000 Genomes Project Japanese population were calculated by LDMAP and plotted against physical distance. LDU for 272 tag SNPs within ~211kb (chr16:13,900,333-14,111,429) region near the *ERCC4*/*XPF* intron variants are present.

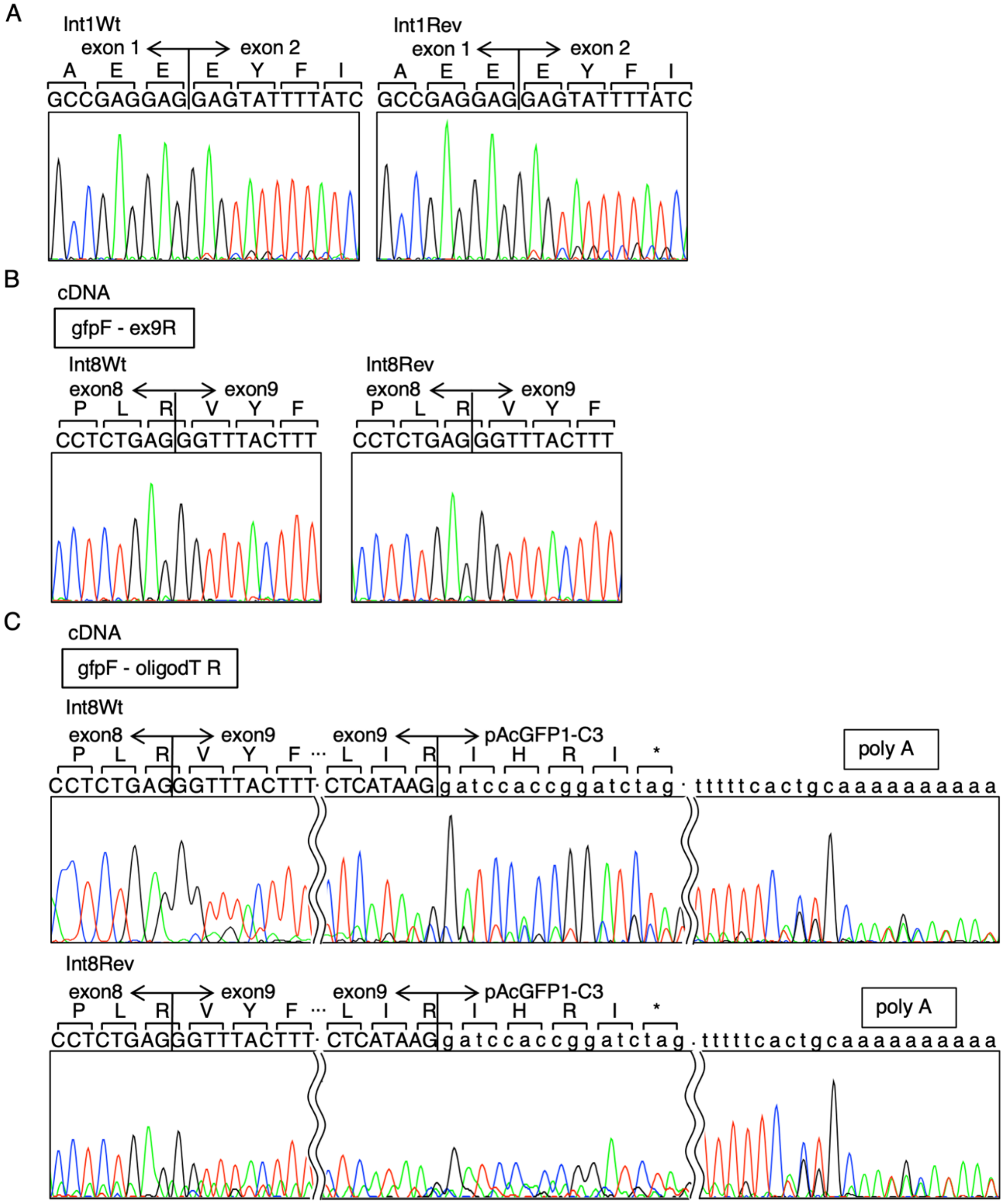

Fig. S3. Aberrant pre-mRNA splicing and alternative polyadenylation events of *ERCC4/XPF* in the cases

(**A**) Direct Sanger sequencing of the cDNA products of exons 1-2 boundary. Int1Wt and Int1Rev gave the same sized normal splicing products. (**B** and **C**) Direct Sanger sequencing of the cDNA products of exons 8-9 boundary (**B**) and the near transcription termination site (**C**). Int8Wt and Int8Rev gave the same sized normal polyadenylated products.

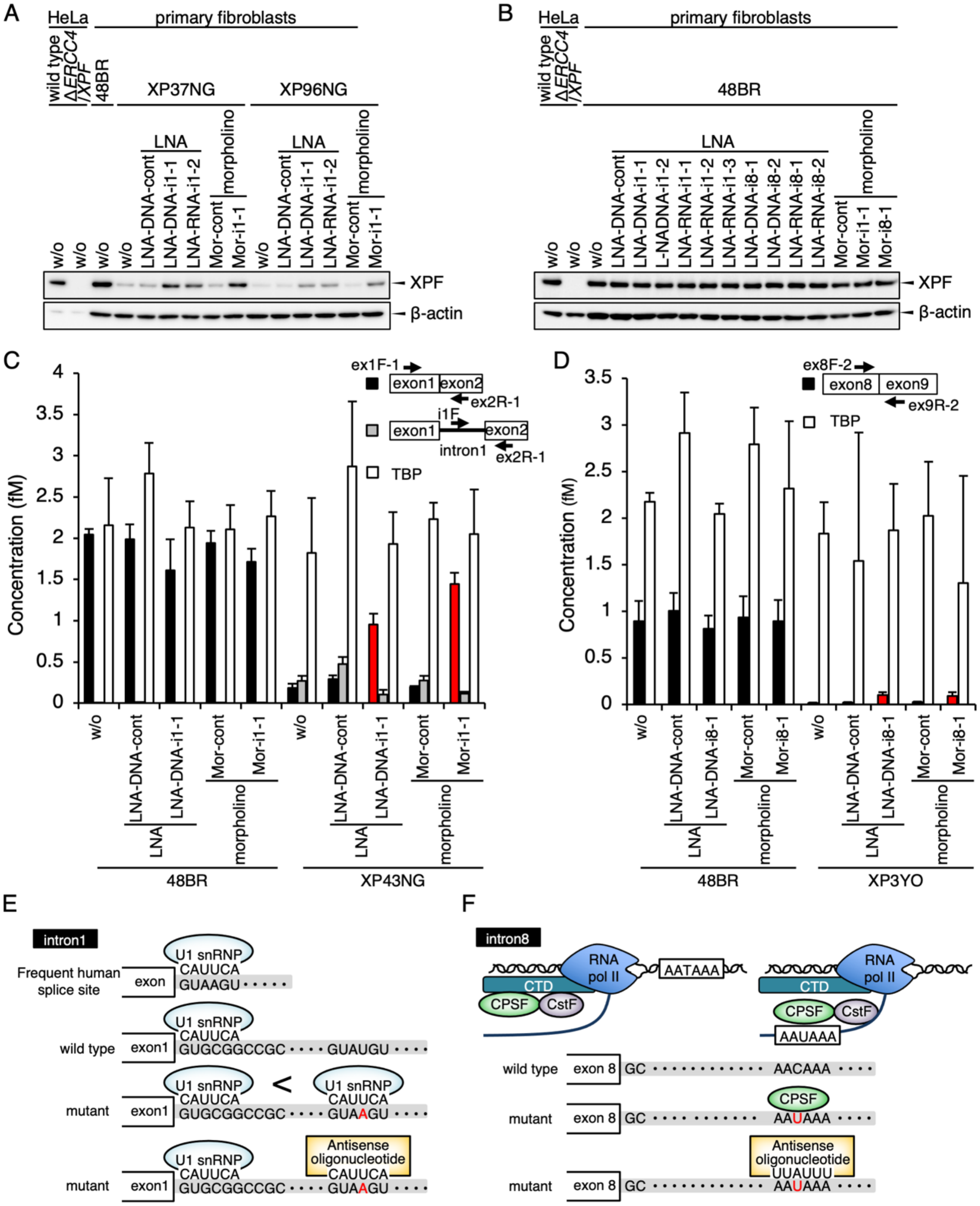

Fig. S4. ASO treatments rescue the *ERCC4*/*XPF* expression
(**A**) Confirmation of the recovery of the XPF protein expression by the top three efficient ASOs. XP37NG and XP96NG, bearing the intron 1 variant were treated with LNA- as well as with morpholino- modified oligonucleotides shown in (**Fig. 4A**). (**B**) The XPF protein expression in normal 48BR was unaffected by the ASO treatments. (**C**) Digital droplet qPCR detected the suppression of the aberrant splicing product of intron 1 (filled bars, PCR amplifying the *ERCC4*/*XPF* exons 1-2 boundary; gray bars, PCR amplifying the 5' cryptic fragment of the *ERCC4*/*XPF* intron 1; open bars, a control PCR product of the *TBP* gene). (**D**) Digital qPCR detected the recovery of the normal polyadenylation products of *ERCC4*/*XPF* (filled bars, PCR amplifying the *ERCC4*/*XPF* exons 8-9 boundary, open bars, *TBP*). Primers used in the qPCR

experiments are listed in **Table S2**. ASOs used in the experiments are listed in **Tables S3** and **S4**. (**E** and **F**) Schematic representation of the 5'cryptic splicing donor site, which is predicted to have a higher binding affinity of U1snRNA than the original sequence (**E**). Similarly, the intron 8 variant results in the polyadenylation consensus sequence (5'-AAUAAA-3'), which is a target for cleavage and polyadenylation specificity factor (CPSF) (**F**).

Table S1. UDS and RRS activities in the studied XP-F patients

Subject ID	UDS	RRS	Complementation group
XP136KO	defective	defective	XP-F
XP37NG	defective	defective	XP-F
XP43NG	defective	defective	XP-F
XP101OS	defective	defective	not tested
XP97NG	defective	defective	XP-F
XP165KO	defective	defective	XP-F
XP103NG	defective	defective	XP-F
XP4NG	defective	defective	XP-F
XP48NG	defective	defective	XP-F
XP90NG	defective	defective	XP-F
XP95NG	defective	defective	XP-F
XP18NG	defective	defective	XP-F
XP133KO	defective	defective	XP-F
XP23OS	defective	defective	not tested
XP96NG	defective	defective	XP-F
XP2YO	defective	defective	XP-F
XP3YO	defective	defective	XP-F

Table S2. Sequences of the PCR primers

ID	Design (5' -> 3')
ex1F-1	CTCCTCTACCACTTTCTCCAGCTG
i1F	CGCGATGACACAGAGAAGGATG
ex2R-1	GAGGGAGGTGTTCAACTCCTTC
ex1F-2	CACTTTCTCCAGCTGCACTG
ex2R-2	CTTCATAGCGACTGTTGCTTGTG
ex7F	CCTAGAAAGCAACCCAAAGTGG
ex8R	CCTGTAGAGCCTCAATAAGAAGGC
ex8F-1	GAGCCAAGATACGTGGTTCTTTATG
ex9R-1	CAAAGCAGTGAGATAGCGTTGTTC
ex8F-2	GAGCTAACCTTTGTTCGGCAGCTTG
ex9R-2	CCGCAAAGCAGTGAGATAGCGTTGTTC
gfpF	CTTCAAGATCCGCCACAACATCGAG
OligodT R	TTTTTTTTTTTTTTTTTTTTVN
TBP ex2F	CCTGCCACCTTACGCTCAG
TBP ex3R	TGGTGTTCTGAATAGGCTGTGG

Table S3. Sequences of the LNA-modified oligonucleotides

ID	Design * (5' -> 3')
LNA-DNA-i1-1	5^c^T^t^A^c^T^t^A^c^G^t^5^t^G
LNA-DNA-i1-2	c^T^t^A^c^T^t^A^c^G^t^5^t
LNA-RNA-i1-1	5^U^U^A^5^U^U^A^C^G^U^5^U
LNA-RNA-i1-2	A^5^U^U^A^C^G^U^5^U^G^U^G
LNA-RNA-i1-3	A^C^5^C^5^U^U^A^5^U^U^A^C
LNA-DNA-i8-1	T^a^A^c^T^t^T^t^A^t^T^a^G^a^A
LNA-DNA-i8-2	a^A^c^T^t^T^t^A^t^T^a^G^a
LNA-RNA-i8-1	A^A^5^U^U^U^U^A^U^U^A^G^A
LNA-RNA-i8-2	U^U^A^U^U^A^G^A^A^A^U^U^A
LNA-DNA-cont	G^t^T^c^A^t^5^c^G^t^A^c^T^t^5

*lower cases, DNA; N, LNA-modified nucleoside; 5, LNA-methylcytidine; ^, phosphorothioate linkages.

Table S4. Sequences of the morpholino-modified oligonucleotides

ID	Design (5' -> 3')
Mor-i1-1	CGACCCCTTACTTACGTCTGTGTTC
Mor-i8-1	CCGAGTCACAGTTATATTAACTTTTATT
Mor-cont	CCTCTTACCTCAGTTACAATTTATA

Table S5. Summary of the off-target profiles calculated by GGGenome

ID	Recovery [*]	No. of predicted off-target sequences[**]			No. of effective off-target sequences	
		Strand (+)	Strand (-)	Total	Region counts[***]	Gene counts[****]
LNA-DNA-i1-1	++	0	0	0	0	0
LNA-DNA-i1-2	+	10	16	26	3	3
LNA-RNA-i1-1	-	10	16	26	3	3
LNA-RNA-i1-2	+	10	15	25	11	11
LNA-RNA-i1-3	-	44	58	102	29	30
Mor-i1-1	++	0	0	0	0	0
LNA-DNA-i8-1	++	24	17	41	15	20
LNA-DNA-i8-2	+	190	172	362	79	84
LNA-RNA-i8-1	-	190	172	362	79	84
LNA-RNA-i8-2	-	323	357	680	145	148
Mor-i8-1	++	0	0	0	0	0

[*]recovery of the XPF protein expression by the ASOs treatments: ++, >50% recovery; +, >20%<50% recovery; -, <10% recovery (the recovery was assessed by measuring the band intensities in Fig. 4A and comparing them to those of the best recovered ASOs).
[**]number of off-target sequences with 1bp mismatch/gap in the original sequences. +/- strand definitions are based on the hg19 (+, 5'->3'; -, 3'->5').
[***]number of ASOs predicted to bind to the sense strand of genic regions.
[****]number of affected genes.
